# Data Mapping From Food Diaries to Augment the Amount and Frequency of Foods Measured Using Short Food Questionnaires

**DOI:** 10.3389/fnut.2018.00082

**Published:** 2018-09-07

**Authors:** Michael Crowe, Michael O'Sullivan, Breige A. McNulty, Oscar Cassetti, Aifric O'Sullivan

**Affiliations:** ^1^Division of Restorative Dentistry and Periodontology, Dublin Dental University Hospital, Trinity College Dublin, Dublin, Ireland; ^2^UCD School of Agriculture and Food Science, UCD Institute of Food and Health, University College Dublin, Dublin, Ireland

**Keywords:** dietary intake assessment, food diary, food frequency questionnaire, short food questionnaire, mapped database, unhealthy food, dental caries, obesity

## Abstract

Collecting accurate and detailed dietary intake data is costly at a national level. Accordingly, limited dietary assessment tools such as Short Food Questionnaires (SFQs) are increasingly used in large surveys. This paper describes a novel method linking matched datasets to improve the quality of dietary data collected. Growing Up in Ireland (GUI) is a nationally representative longitudinal study of infants in the Republic of Ireland which used a SFQ (with no portion sizes) to assess the intake of “healthy” and “unhealthy” food and drink by 3 years old preschool children. The National Preschool Nutrition Survey (NPNS) provides the most accurate estimates available for dietary intake of young children in Ireland using a detailed 4 days weighed food diary. A mapping algorithm was applied using food name, cooking method, and food description to fill all GUI food groups with information from the NPNS food datafile which included the target variables, frequency, and amount. The augmented data were analyzed to examine all food groups described in NPNS and GUI and what proportion of foods were *covered, non-covered*, or *partially-covered* by GUI food groups, as a percentage of the total number of consumptions. The term *non-covered* indicated a specific food consumption that could not be mapped using a GUI food group. “High sugar” food items that were *non-covered* included ready-to-eat breakfast cereals, fruit juice, sugars, syrups, preserves and sweeteners, and ice-cream. The average proportion of consumption frequency and amount of foods not covered by GUI was 44 and 34%, respectively. Through mapping food codes in this manner, it was possible, using density plots, to visualize the relative performance of the brief dietary instrument (SFQ) compared to the more detailed food diary (FD). The SFQ did not capture a substantial portion of habitual foods consumed by 3-year olds in Ireland. Researchers interested in focussing on specific foods, could use this approach to assess the proportion of foods *covered, non-covered*, or *partially-covered* by reference to the mapped food database. These results can be used to improve SFQs for future studies and improve the capacity to identify diet-disease relationships.

## Introduction

Exploring potential diet-disease relationships requires an accurate estimate of food intake. The difficulties associated with measuring diet are well documented (1–5). Collecting accurate and detailed dietary intake data is costly at a national level, and so dietary assessment tools are often modified or limited accordingly (2, 6). While all dietary assessment methods are prone to measurement error (3, 7) there are a number of factors to consider when selecting the most appropriate method, particularly for young children where the primary caregiver (PCG) usually provides a proxy report of food intake (8). Firstly, it is important to consider which aspect of the diet is of interest such as specific foods, episodically consumed foods, or total food and nutrient intake, while study design and objectives will also impact the method selected. In large-scale cohort surveys dietary intake is often assessed to either describe usual intake distributions or estimate the relationship with a particular health outcome.

Food Frequency Questionnaires (FFQ), 24 h recalls, multiple-day food diaries (FD) or records, diet histories, and biomarkers are some of the most commonly used methods to assess dietary intake (1, 3, 6, 9, 10). Smaller studies tend to use prospective methods such as the detailed weighed FD over a number of days or weeks which can estimate the distribution of habitual intake of a food group (9). Despite having limitations, the weighed FD method is considered the “standard” reference for relative validation in nutrition research (5, 11). In addition to the self-reported methods described, there are a number of dietary biomarkers that reflect nutrient and food intakes; for example, serum vitamins, blood lipids, and urinary metabolites (12, 13). Comprehensive reviews of the different methods, their limitations, and strengths have been widely reported (1–5, 10, 14).

A FFQ is the most widely used dietary assessment method for epidemiological studies and this is sometimes further modified in terms of time-frame, food items, and estimation of quantity (6). However, the data generated is limited, particularly if key foods are omitted and minimal consumption frequencies recorded. Even relatively simple descriptive analysis of “unhealthy” food intake data can be compromised and bias our understanding of the potential association with chronic disease (3, 15). Furthermore, measuring habitual food intake has a number of inherent issues such as self-selection and social desirability bias, and selective underreporting of specific foods (3, 8, 16). Short Food Questionnaires (SFQs) are increasingly used in national cohort surveys to measure aspects of dietary intake, however, publications rarely report details of relative validation or measurement error (6).

Following dietary data collection, food consumption must be linked with food composition tables to determine nutrient and food group intakes for a given population. FAO/INFOODS developed a set of guidelines to achieve the most appropriate food matching (17). Accurate food matching is critical to obtain high quality estimates of nutrient intakes (17, 18). The FAO/INFOODS protocols highlight key fields to consider such as identifying the food component of interest, food name and descriptors, and identifying the characteristics of the population of interest (17). Automatic or semi-automatic methods have also been proposed to improve the speed and scale of mapping FD to food composition tables (18). Similar methods have been developed to automatically map FFQ data directly from the questionnaire to food composition tables (19). Ultimately, while further harmonization of food composition tables is desirable to convert dietary records and generate good quality data for nutrition and epidemiological research, the type of dietary assessment method used may restrict the research potential of the data captured (17). Linking SFQ data to food composition tables is problematic, especially when there are a limited number of food groups recorded. Alternatively, where a matched cohort exists, it is possible to link the SFQ data from the large cohort to more detailed food intake records collected from the matched cohort. This approach could improve the research potential of the SFQ data and provides the means to assess the performance of the SFQ relative to an accepted standard.

An “unhealthy” diet is a major factor that contributes to obesity, diabetes, cardiovascular disease, and poor oral health (20, 21). Sugar containing foods and drinks are targeted as a means to reduce total energy intake and therefore help control body weight and obesity (22–25). Sugar intake is also the most important risk factor for dental caries (26). Therefore, it makes sense to take a common risk factor approach to address both conditions, given limited public health resources (26–28). Recent studies have also indicated that the preschool period is a critical opportunity for early intervention to promote healthy growth, body composition, and dental health (29, 30). In particular, studies show that early changes in dietary behavior can result in remineralisation of non-cavitated early lesions in teeth (31, 32). Similarly, although there is a paucity of studies at this age, multicomponent programs to prevent or treat childhood obesity, particularly with parental involvement, have successfully impacted on preschool child weight (33). However, despite common linkages between obesity and dental health, and the evidence to support early intervention in both cases, very few studies report anthropometrics, dental indices, and good quality dietary data, particularly for preschool aged children (29, 30).

Growing Up in Ireland (GUI) is a nationally representative longitudinal study of infants in the Republic of Ireland. The cohort was recruited initially in 2008 when infants were 9 months old, with repeat collections at age 3, 5, and currently at age 9 years. A SFQ (with no portion sizes) was used to assess the intake of “healthy” and “unhealthy” food and drink when GUI children were 3 years old (34, 35). Parents were asked to report consumption of 15 food groups when completing a researcher-led questionnaire. The National Preschool Nutrition Survey (NPNS) is a cross-sectional dietary intake survey designed to assess the habitual food and drink consumption of a nationally representative sample of children aged 1–4 years (36). The NPNS used a detailed 4 days weighed FD to record food and drink intake which included 3 researcher visits for training and data checking. Both studies collected data from 3 year old children in Ireland in 2010–2011.

In this paper, we describe a method that can be used to link matched datasets from two studies to improve the quality of dietary data collected using SFQs in large cohort surveys. We apply this method using two national surveys that collected dietary data from 3 year old children: (i) GUI which collected food consumption data using a SFQ and (ii) NPNS which collected food consumption data using a weighed FD. We report foods that were *covered* or *non-covered* by the SFQ in GUI relative to the detailed dietary assessment in NPNS. We focus in on high-sugar foods to illustrate the potential implications of using limited SFQs for epidemiological research. In this study the NPNS food database was used as the “reference standard” to map onto the larger national cohort survey and create an augmented food intake database (37). This study adds to previous reporting of the risk involved when selecting brief SFQs for large studies which may be less costly and less burdensome than detailed methods but increase the risk of attenuating the relationship between dietary factors and health outcomes (2, 6, 10, 11).

## Methods

### Data collection and participants

This research used data collected as part of two studies: the second wave of the GUI infant cohort longitudinal survey which was carried out by the joint Economic Social Research Institute-Trinity College Dublin (ESRI-TCD) GUI study team from December 2010 to July 2011 and the NPNS cross-sectional study which was conducted by Irish Universities Nutrition Alliance (IUNA) from October 2010 to September 2011. The second wave of the GUI infant cohort were 3 years of age at the time of interview (*n* = 9,793). The NPNS had a total sample of 500 children aged 2–4 years; but only the 3-year olds were included for this analysis (*n* = 126). Both samples were nationally representative, and surveys were conducted at a similar time. GUI selected a random sample, on a systematic basis, pre-stratified by marital status, county of residence, nationality, and number of children from the National Child Benefits Register which is a universal welfare entitlement in the Republic of Ireland (38). NPNS used a quota sampling approach to obtain a sample of 125 children within each of the four preschool age groups between 1–4 years of age (39). The NPNS sample was recruited from an Irish parenting resource database (https://www.eumom.ie/) or from childcare facilities randomly chosen in selected locations (36).

GUI-trained fieldworkers completed the interview with the PCG, after consent was obtained, in the family home using a computer assisted personal interview (CAPI). The PCG was defined as the person, in most cases the mother and biological parent, who delivered most care to the study child and was best placed to provide any relevant information about him/her in response to the survey questionnaire administered. Full details of the population, sample design, participant response, fieldwork/implementation, survey instruments, structure and content of the datafile, and interviewer training are available from GUI at http://www.esri.ie/growing-up-in-ireland/ (35, 38). In the NPNS study the researcher visited the participant's home on three occasions during the 4 days food record period. Full details for NPNS are available at http://www.iuna.net/ (36). These include details of the quality procedures that were used to help consistency and minimize error throughout the collection and manipulation of the food intake data. The CAPI questionnaires used in GUI mainly used closed questions. The program incorporated an extensive range of cross-variable consistency checks (38). The Anonymised Microdata Files (AMF) for GUI are available as flat rectangular datafiles (SPSS format) on application to the Irish Social Science Data Archive (ISSDA, UCD, Dublin). Access to the more detailed Researcher Microdata File (RMF), which were used for this study, is subject to appointment of the researcher as an Officer of Statistics by the Central Statistics Office. The NPNS datafiles are available on application to IUNA.

Both studies were conducted according to guidelines laid down in the Declaration of Helsinki. Ethical approval for the GUI project was received from a Research Ethics Committee convened by the Department of Health and Children while approval for the IUNA-NPNS project was obtained from the University College Cork Clinical Research Ethics Committee of the Cork Teaching Hospitals, University College Cork.

### Food intake measurement

In the GUI study, dietary intake was assessed using a SFQ, previously used in the Longitudinal Study of Australian Children (LSAC), to characterize healthy and unhealthy food intake (34). The PCG reported how frequently their child consumed 15 food categories during the previous 24 h. Intakes were recorded as once, more than once, or none at all. No information on food portion size was recorded. Foods were categorized as “healthy” or “unhealthy.” The healthy food groups included: fresh fruit, cooked vegetables, raw vegetables or salad, full fat cheese/yogurt/fromage frais, low fat cheese/low fat yogurt, full cream milk or full cream milk products, skimmed/semi-skimmed milk or milk products, and water (tap, still sparkling). The unhealthy food groups included: hamburger, hot dog, sausage, meat pie, hot chips or french fries, crisps or savor snacks, biscuits, doughnuts, cake, pie or chocolate, sweets, fizzy drinks/minerals/cordial/squash (diet), fizzy drinks/minerals/cordials/squash (not diet). The GUI FFQ is available at: http://www.ucd.ie/issda/data/growingupinirelandgui/.

A 4 days weighed food record was used in NPNS to collect food and beverage intake data (36). At least one of the 4 days included a weekend day and a nutrition researcher trained the caregivers on how to use the FD and weighing scales to record intakes. The caregivers were requested to record information relating to the amount, brand and type of foods, and beverages consumed by the child and to include cooking method, recipes, packaging type, food leftover and time of eating occasion. Food and beverage intake data were reported after weighing, in grams. The protocol used for quantification and nutrient intake estimation is available at http://www.iuna.net/ and has been previously reported (39). In total, there were 1,652 different food codes in the NPNS and each food was also assigned to one of 77 food group categories.

### Data preparation and mapping protocol

**Figure 1:**
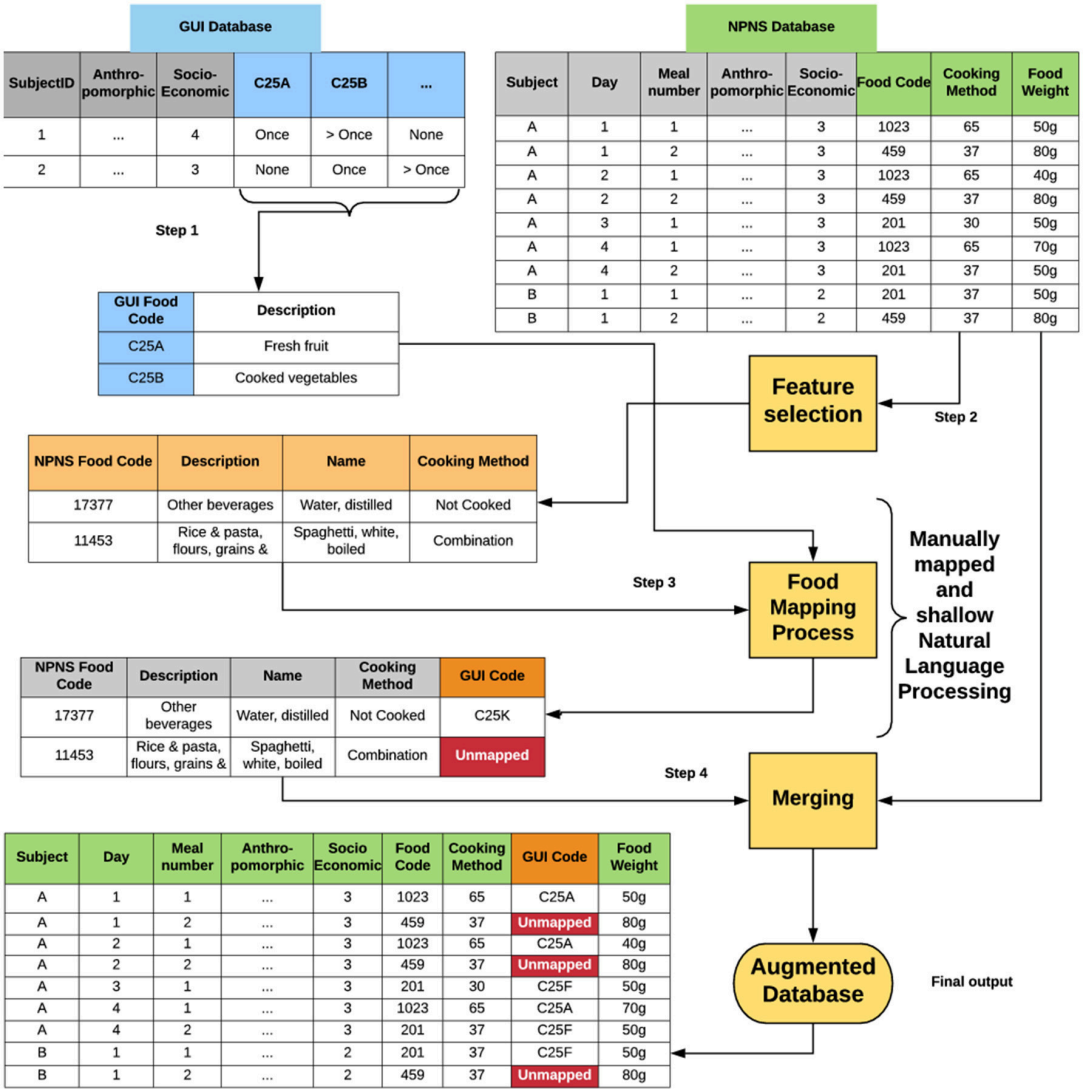
Flow diagram showing data processing steps for unidirectional mapping of GUI food codes with NPNS food codes. Step 1: feature selection from GUI database; Step 2: feature selection from NPNS database; Step 3: mapping process; Step 4: Merging of databases following mapping process. GUI, Growing Up in Ireland; NPNS, National Preschool Nutrition Survey. Feature selection identified variables from both GUI and NPNS databases that were desired, e.g., socioeconomic class, cooking method, food weight. All GUI codes were manually mapped with food categories from NPNS, e.g., NPN food code 17377 mapped to GUI code C25k; NPNS food code 11453 was unmapped and this created a *non-covered* food group.

**Figure 2:**
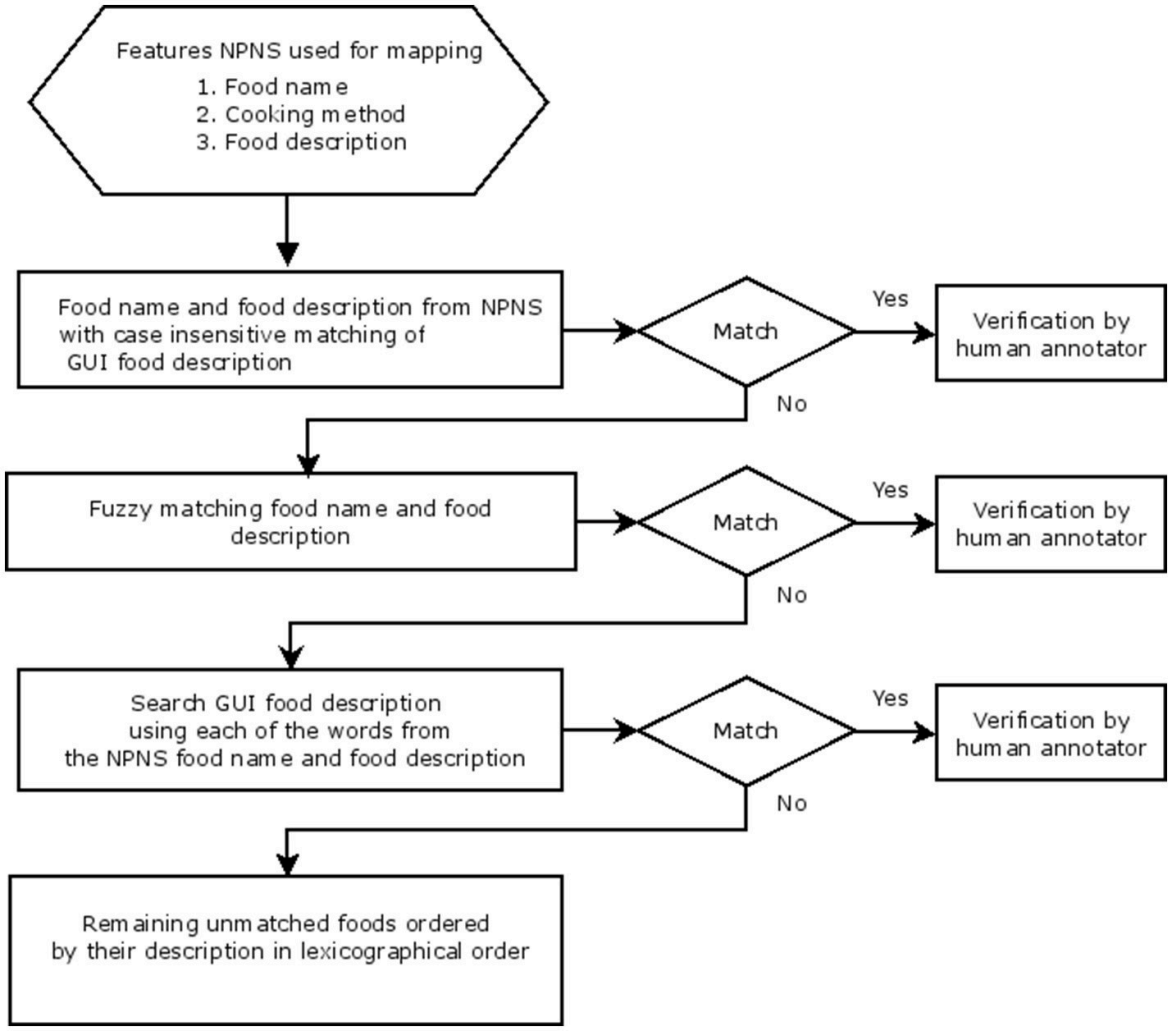
Decision Algorithm for mapping of GUI food codes with NPNS food codes indicating the stepwise protocol used. A diamond indicates a decision (match: Yes or No) and a rectangle indicates the process used at each step for mapping or verification by human annotator.

Data files were imported from SPSS (v. 20.0: SPSS, Chicago, IL) or converted to.csv format before importing to R (version 3.2.2) for linkage and analysis. The 77 Food group categories described in the NPNS dataset were used for this analysis and other variables such as food name, cooking method, day of consumption, meal-type, and food description were also selected (Supplementary Material). All food categories in NPNS were sorted, grouped and filtered to facilitate easy mapping. A unidirectional mapping procedure (Figure 1) was carried out using a manual mapping and shallow natural language processing (NLP). This involved a stepwise protocol (Figure 2) using direct food name/food description matching, fuzzy matching, or word search using each word of the NPNS food name/food description. Each step was verified by a human annotator. A second human annotator applied the same protocol to repeat the mapping and compare the repeatability of the method. Finally, the results were checked, independently, by a nutritionist. All GUI food groups were filled with information such as frequency and amount of food, anthropomorphic status, meal type, and social class from the NPNS food datafile and consolidated into a single augmented database.

The augmented data were analyzed to examine all food groups described in NPNS and GUI and what proportion of foods were *covered, non-covered*, or *partially-covered* by GUI food groups relative to the NPNS database which included a more detailed dietary record. The term *non-covered* indicated a specific food consumption that could not be mapped using a GUI food group, i.e., the food in NPNS was not matched by the same food in GUI. A food consumption was described as *covered* if there was a matching GUI food group that the food consumption could be mapped to, i.e., the food in NPNS was matched by the same food group in GUI. A consumption in NPNS was defined as any eating occasion (EO) of a food or drink (snack or main meal) and an entry in the food diary was considered a consumption.

### Quantitative analysis of mapped data and augmented database

The initial aggregation was completed at the subject and survey day levels. Aggregate metrics were defined and determined for all food items included mean, interquartile range, maximum, minimum, standard deviation, and standard error of the mean. Aggregates estimated included the frequency and amount (g/day) of *covered, non-covered*, and *partially- covered* food groups which were also expressed as a percentage of the total amount of food consumed (Supplementary Material). Our analysis treated each day of the 4 days in NPNS as an independent day. The mean daily intake amount (g/day) and the frequency of each food consumed was calculated for each NPNS participant, by summing the amount of all food consumptions a subject consumed by food group, averaging across the 4 days for each subject and then calculating the total sample average. Frequency was estimated by summing the total number of times the food appeared in the diary and dividing by four, i.e., the number of days in the survey.

Estimates were also derived for the percentage of consumptions per subject per day for each NPNS food group that was *non-covered* as a percentage of the total number of consumptions. A similar ratio was calculated for the percentage amount of food items *non-covered* over the total amount of food consumed per day. The SFQ used in GUI could not estimate habitual intake over time, and therefore will contain systematic error due to the lack of detail compared to the 4-day weighed food diary and did not capture infrequently consumed foods (3, 15). As some food codes in NPNS were partially mapped by GUI the% of coverage was estimated for all foods.

**Figure 3:**
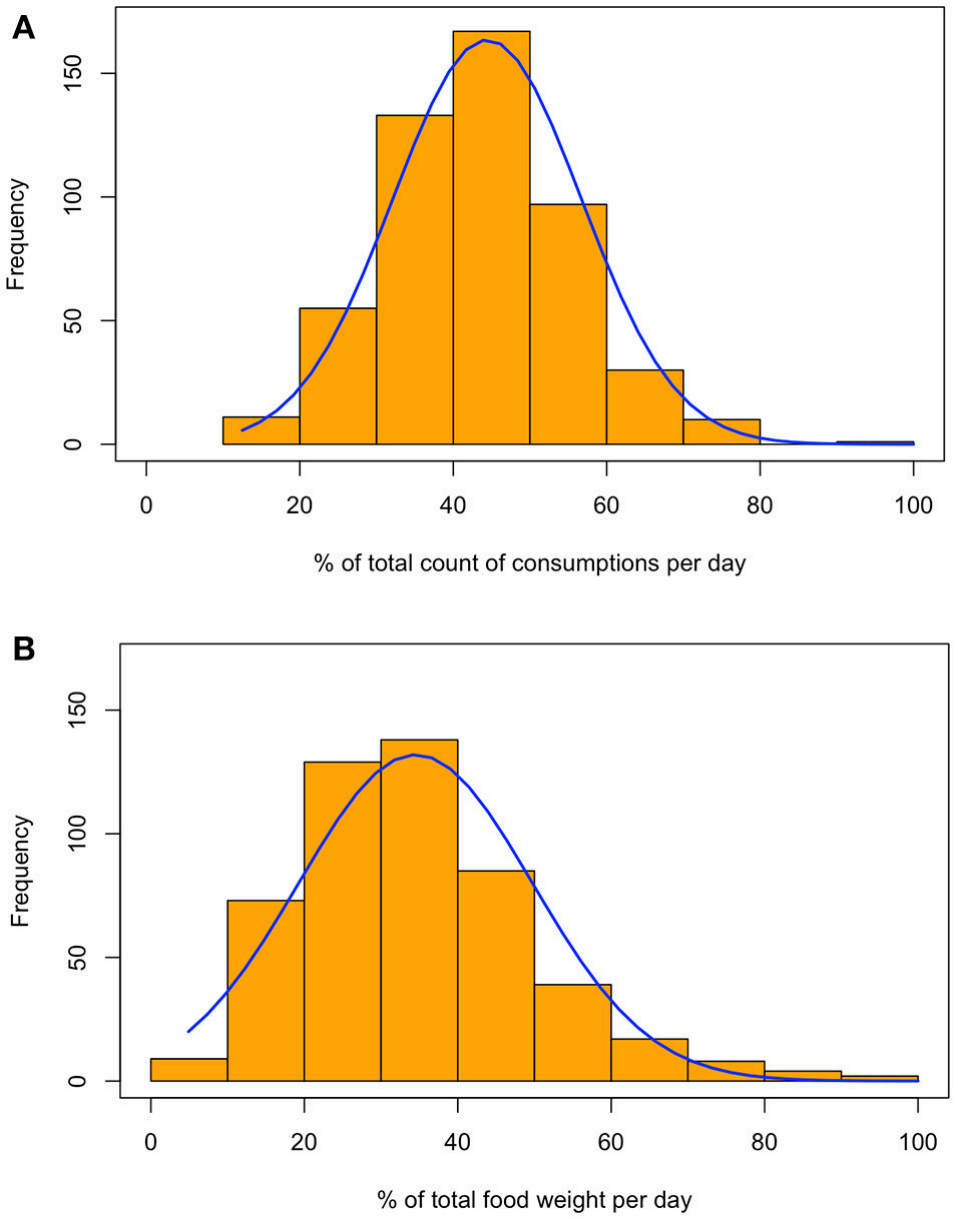
Food frequency and consumption weight *non-covered* by GUI survey representing the distribution of the ratio of consumption counts **(A)** or weight **(B)** of a food item consumed in NPNS that were *non-covered* by the mapped GUI data model.

The total number of times when a *non-covered* food was consumed (total consumption frequency per day) and the total food amount (g/d) of a *non-covered* food was calculated. The ratio of the frequency of consumption of *non-covered* food over the total food frequency was determined. A similar ratio was determined for *non-covered* food consumed over amount of total food consumed. The frequency distributions of the ratio of consumption frequency and amount of *non-covered* food consumed divided by the total food consumed were displayed as histograms. Using a non-parametric density estimation the distribution of the proportion of non-covered food was displayed graphically (Figures 3A,B) and tested formally using a permutation test, the Wilcoxon rank sum test (*p* < 0.01) (40).

## Results

A unidirectional mapping protocol (Figure 1) created an augmented food database which was then aggregated to produce quantitative metrics to assess how well the SFQ in GUI performed in matching a detailed national food database for the same age cohort in NPNS. Characteristics of both the NPNS and GUI surveys are presented in Table 1. When the mapping by two separate human annotators were compared the results were the same except for one single food code. This was then remapped following a decision by the nutritionist. The frequency and amount of food consumed that was not mapped by the GUI survey is depicted in Figures 3A,B, respectively. The histograms represent the distribution of the ratio of consumption counts (Figure 3A) or amount (Figure 3B) of food items consumed per person per day in NPNS that were not covered by the mapped GUI database divided by the total number of consumptions or amount, respectively, per day. For example, the ratio of consumption counts is the number of food consumptions *non-covered* by the mapped GUI model divided by the total number of food consumptions in any given day. The overall pattern of the distribution of percentage consumption frequency was symmetrical while the shape of the distribution for percentage food amount was skewed slightly to the right. The mean (SD) for consumption frequency was 44% (12%) and for consumption amount was 34% (15%). As some food codes in NPNS were partially mapped by GUI the % of coverage was estimated for all foods. For example, other fruit in NPNS was partially mapped to GUI and ~ 63% of this food group was *non-covered*.

**Table 1:** Comparison of survey characteristics of National Preschool Nutritional Survey (NPNS) and Growing Up in Ireland (GUI) national infant cohort survey.

	**NPNS**	**GUI**
Sample size (*n*)	126	9,793
Subject age	3 years	3 years
Nationally representative	Yes	Yes
Date of survey	Oct 2010–Sept 2011	Dec 2010–July 2011
Food measurement instrument	4 days weighed food diary	Short food questionnaire

**Figure 4:**
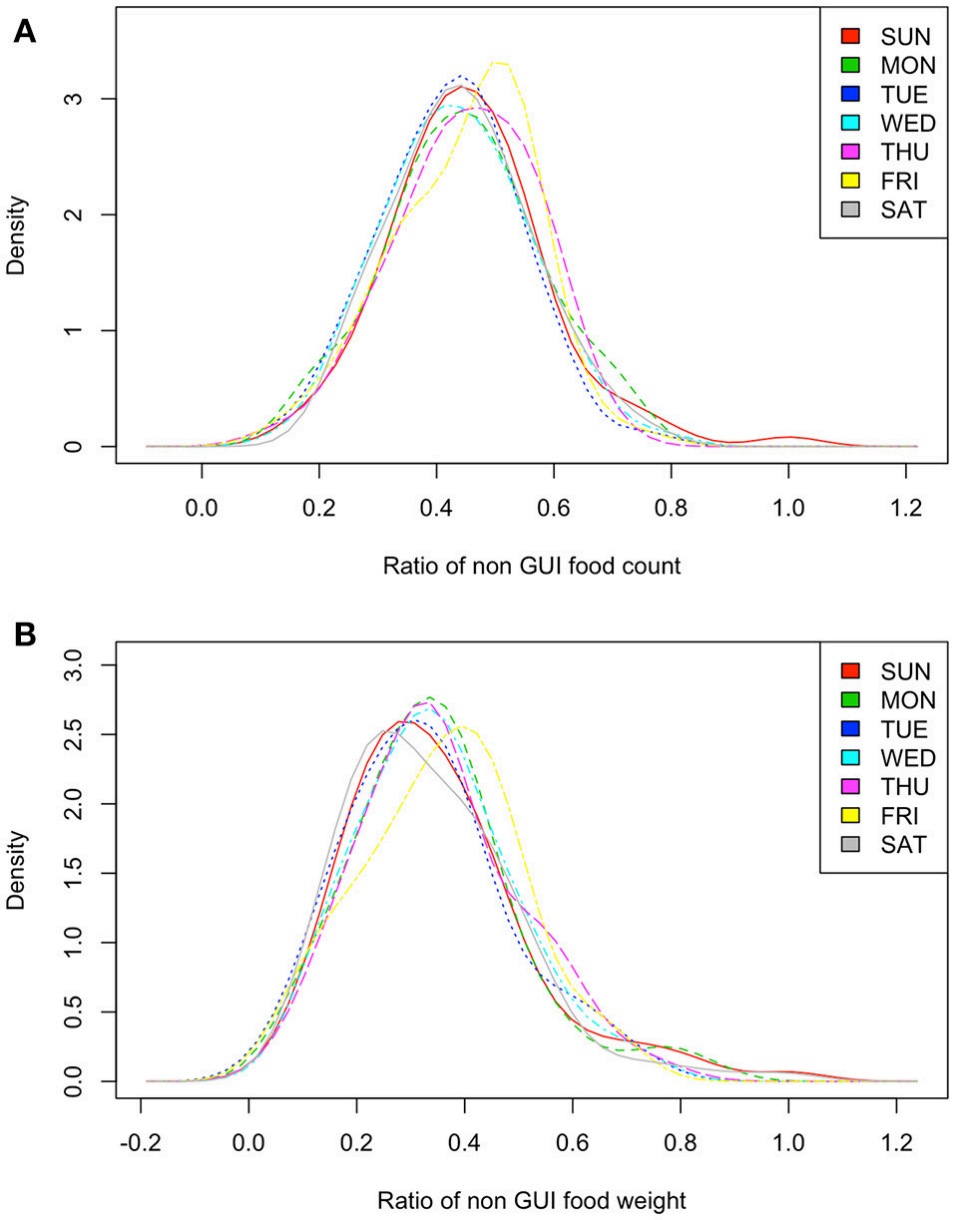
Food frequency and consumption weight *non-covered* by GUI survey by the day of the week representing the distribution of the ratio of consumption counts **(A)** or weight **(B)** of a food item consumed in NPNS that were *non-covered* by the mapped GUI data model over the total food *covered*.

A selection of the most commonly consumed *non-covered* (by GUI) food items during the NPNS 4-day period is displayed in Table 2. Food items rich in sugar that were *non-covered* included ready-to-eat breakfast cereals, fruit juice, sugars, syrups, preserves and sweeteners, and ice-cream. The distribution of the proportion of *non-covered* food (frequency of consumption and amount, g/d) by the day of the week is displayed in Figures 4A,B as density estimates. The distribution of the ratio of *non-covered* food to total food varied according to the day of the week. The distribution patterns on Friday and Sunday appeared to have some differences from the other days with a shift of the distribution to the right for Friday and an increased “tail” on Sunday. Permutation tests were carried out which omitted 1day from each test while retaining all the others which suggested that the distributions for each day of the week were significantly different from each other, *p* < 0.01 (except Monday for consumption frequency and Sunday and Monday for consumption amount).

**Table 2:** Number of Eating Occasions (EO), Food amount (g/day) and Standard Deviation (SD) of selected *non-covered* food items in augmented food database.

**Food group description**	**EO number**	**Food amount (g/d)**	
		**Mean**	***SD***
Ready-to-eat breakfast cereals	351	31	17
White sliced bread and rolls	239	61	34
Other spreading fats	224	8	5
Wholemeal and brown bread, and rolls	192	50	29
Fruit juices	190	173	123
Soups, sauces, and miscellaneous foods	190	52	71
Potatoes	163	79	46
Sugars, syrups, preserves, and sweeteners	158	12	13
Bacon and ham	148	30	25
Rice and pasta, flours, grains, and starch	131	86	57
Supplements	124	106	60
Meat products	95	52	43
Butter	90	9	8
Ice creams	89	57	25
Beef and veal dishes	88	129	82
Chicken, turkey, and game	87	44	26
Other breakfast cereals	83	130	83
Eggs and egg dishes	64	60	30
Fish and fish products	62	62	32

## Discussion

Substantial progress has been made in assessing and interpreting dietary intake data (5, 11). However, as emphasized in a recent systematic review (6) there is a need to provide guidance on which questions to use to measure children's food intake and this will depend on the research focus and study sample. The aim of this analysis was to develop a mapping procedure that allowed detailed dietary data from a matched cohort to be mapped to simple data from a large cohort with the aim of improving the quality of dietary data in large cohorts and therefore improve the capacity to identify diet-disease relationships. In doing so, it was possible to evaluate the performance of a SFQ compared to the “gold standard” FD for estimating food and nutrient intakes. Our protocol was developed to allow for manual mapping of a SFQ using the food description and cooking method from the more detailed FD to link two different datasets. As well as making code in R Markdown available a future possibility would be to automate the procedure by using a machine learning classifier combined with fuzzy matching to refine the mapping of difficult items (18). This would reduce the time burden, human error risk, and provide a fully reproducible process (18, 41).

Rather than report the average weight or frequency of food consumed the proportion of these metrics as a percentage of the total food consumed was estimated to illustrate how much of the foods from the detailed NPNS were *covered* or *non-covered* by the SFQ used in the GUI survey. As illustrated in Figure 3, there was a wide spread of the distribution and the mean (SD) for consumption frequency of foods not covered by GUI was 44% (12%) and for consumption amount was 34% (15%). Thus, the SFQ in GUI did not capture a substantial portion of habitual foods consumed 3-year-olds in Ireland. When evaluating the relative validity of any dietary assessment tool it is important that the test method and reference method measure the same underlying concept over the same time period (6). The approach here was to use the reference method (4-day weighed FD) to map the food groups in the test method (SFQ). The SFQ used in GUI was not designed to capture habitual food intake but reflected what is often used in large scale-interdisciplinary surveys.

While SFQs are more likely to be reliable than accurate (6) there is less detailed knowledge of the type of measurement error with SFQs than other more detailed instruments (9). SFQs are obviously appealing to include in a survey to measure dietary intake, but these brief screeners tend to be widely used without relative validation (6, 9, 15). The lack of accurate estimates of dietary intake may lead to biased determinations of relationships between for example, consumption of “unhealthy” food and overweight/obesity or, sugar consumption and dental caries. Other researchers have highlighted the benefits of combining information from multiple surveys to gain and augment estimates of parameters lacking in individual surveys (42, 43).

While this data analysis was carried out retrospectively these results highlight the importance of selecting the most appropriate dietary assessment instrument given the study design, resources, and objectives. However, the protocol described here could be applied in other scenarios, particularly *post-hoc* interdisciplinary studies to link datasets for further analysis. Where knowledge of habitual dietary intake is required it may be possible to plan the alignment of a national cohort with a similar population sample nutritional survey to maximize the value of data extraction. The use of data linkage and other techniques such as integrated health modules within longitudinal surveys should be explored.

Inappropriate feeding patterns of “unhealthy” or “sugar rich” food and drink appear to start as young as 6 months of age (30, 32, 44) and tend to increase as the child moves to solid foods in the first few years of life (45). It would appear reasonable to use a SFQ focussed on capturing “unhealthy” food intake in a national cohort survey. However, the results presented here highlight the lack of capture of some foods and drinks rich in sugar (Table 2) and commonly implicated in causing dental caries. As analyses of large cohort child surveys are commonly used to inform key public health policy related issues such as oral health or childhood obesity services it is important that appropriate dietary information can be extracted to maximize the full potential of these studies. As well as the lack of appropriate questions in the SFQ to capture these items, day-to-day variation can also contribute to insufficient estimation particularly as habitual intake of food and drinks rich in added sugar has been reported to be higher at weekends compared to weekdays (46). In this analysis, some differences were noted in the distribution of both amount and frequency of consumption of *non-covered* food on Friday and Sunday compared to other days of the week (Figure 4) but most days of the week showed significant differences from each other using a permutation test.

### Strengths and limitations

Although the results highlight key shortfalls of the SFQ, it is important to acknowledge that the GUI survey was not designed to report detailed dietary intakes *per se* but to use a brief screener-type SFQ which collapsed food groups into what was considered “healthy” and “unhealthy.” The categorisation could potentially introduce bias as PCGs may under or over report due to social desirability of what is perceived as “healthy” and “unhealthy” foods. Compared to other food mapping algorithms such as free sugar estimation (47) the mapping protocol in this analysis contained a low risk of subjectivity as the degree of detail included (e.g., cooking method and detailed food description) facilitated accurate mapping to match the GUI food codes. However, the manual mapping procedure is time-consuming and is subject to a risk of human error. Although both sample cohorts were closely matched there is a risk of bias from multiple sources including slight differences in time periods when the surveys were carried out, differences in under/over-reporting by PCGs and differences in the day of surveying.

## Conclusion

This data analysis protocol provides a method for further mapping of national cohort surveys and food databases for other age cohorts. Through mapping the food codes in this manner and estimating the degree of *non-covered* food it was possible to visualize the relative performance of the brief dietary instrument (SFQ) compared to the more detailed one (FD) especially in capturing specific food types. The SFQ did not capture a substantial portion of habitual foods consumed by 3-year-olds in Ireland. Researchers interested in focussing on specific foods, such as those rich in sugar, could use the methods described here to assess the proportion of foods *covered, non-covered*, or *partially-covered* by reference to the mapped food database. Using this approach to successfully map datasets will help improve SFQs for future studies and improve the quality of the data that can be extrapolated, therefore improving the capacity to identify diet-disease relationships.

## Author contributions

MC, AO, OC, and MO contributed to the study design and interpretation of results. MC and OC performed the data analysis. MC, AO, and MO prepared the manuscript. BM contributed to the design and execution of the NPNS data collection. All authors read and approved the final manuscript.

### Conflict of interest statement

The authors declare that the research was conducted in the absence of any commercial or financial relationships that could be construed as a potential conflict of interest.
