# Supplementary material for: Data Mapping From Food Diaries to Augment the Amount and Frequency of Foods Measured Using Short Food Questionnaires
Source: Front Nutr. 2018 Sep 7;5:82. doi: 10.3389/fnut.2018.00082 (PMC6190565; doi:10.3389/fnut.2018.00082)
Supplement: Supplementary file 1 [file Data_Sheet_1.PDF]

1. IUNA NPNS mapped to GUI. Rmd

Key packages

- `SQLDF` to join data: joins data more effectively using SQL (link to http://www.iana.org/assignments/media-types/application/sql)
- `foreign` read the SPSS data

```
library(sqldf)

'foreign' was built under R version 3.4.1

storePath <- "tmp"
```

NPNS data was in two separate files

1. Anthropometric data
2. Food diary

Joined both files as needed the anthropometry data in the analysis

Join is on subject id

```
# Read the two datasets
antropoDf <- read.csv("npns-antropometry-data.csv", header = TRUE)
fooddataDf <- read.csv("npns-derived-v1_copy.csv", header = TRUE)

# Join the two dataset using the id of the subject
foodDataAgeDf <-
sqldf("SELECT f.*, a.* FROM fooddataDf f LEFT JOIN antropoDf a
ON f.SUBJECID = a.ID")
```

Limited to 3 years old

```
# Looking at only 3 and 5 years old - Note there is no 5 years subjects
foodDataForMapping <- sqldf("SELECT * from foodDataAgeDf WHERE AGE = 3")
```

The GUI food questionnaire and the NPNS data in SPSS format

```
# Reading in the GUI codes
guiFoodQuestions <- read.table(file="GUI-qn.txt", sep=".")
colnames(guiFoodQuestions) <- c('code', 'description')
foodDataSPSS <- read.spss(file="npns-food-file-4R.sav")

## re-encoding from CP1252
```

Included the cooking methods description to build the dataset that is used for mapping using the following variables

3. Food code
4. Food description (short and long)
5. Cooking method (code and description)
6. F77 food category

```r
# Loaded initially to map against the 77 food categories
iunaFoodCodes <-
cbind.data.frame(code = seq(1, 77),
description = levels(foodDataSPSS$IUNA_NPNS_77FG))

# Loaded initially to use the cooking
cookingMethodsCodes <- read.table(file = "CMETH.txt", sep = "=")

colnames(cookingMethodsCodes) <- c('code', 'description')

# Group diary entries by IUNA Code 77 and cooking method.
foodGroupsCmeth <-
sqldf("SELECT IUNA_NPNS_77FG, CMETH, count(*) ct FROM foodDataForMapping G
ROUP BY 1,2 ")

# Add description to the cooking method
foodGroupsCmethExt <-
sqldf(
"SELECT fc.description FOODNAME, cm.description COOKINGMETHOD , fg.*
FROM foodGroupsCmeth fg
LEFT JOIN cookingMethodsCodes cm ON cm.code = fg.CMETH
LEFT JOIN iunaFoodCodes fc ON fc.code = fg.IUNA_NPNS_77FG"
)


write.csv(
foodGroupsCmethExt,
file = paste(storePath, "iuna-food-groups-cooking-methods.csv", sep = "/
"),
row.names = FALSE
)

# List of all distinct food in the diary
allFoods <-
sqldf(
"SELECT distinct IUNA_NPNS_77FG, CMETH, FCODE, Food_description_first_firs
t
FROM fooddataDf  "
)
## IUNA_NPNS_77FG is too coarse we need to use the actual food codes


allFoodsExt <-
sqldf(
"SELECT fc.description FOODNAME, cm.description COOKINGMETHOD , fg.*
FROM allFoods fg
LEFT JOIN cookingMethodsCodes cm ON cm.code = fg.CMETH
LEFT JOIN iunaFoodCodes fc ON fc.code = fg.IUNA_NPNS_77FG"
)

write.csv(
```

```
allFoodsExt,
file = paste(storePath, "iuna-food-allFoods-with-groups.csv", sep = "/")
,
row.names = FALSE
)
```

Mapping done in Google sheet with filters

At this point the mapping was read. If not mapped we set the `GUI_CODE` to `NULL`

```
mappings <- read.csv(file=paste("iuna-gui-mapping-2015-12-21.csv", sep="
/"), header = TRUE)

# Setting all empty i.e non categorized foods to NA
mappings[mappings$GUI_CODE == "",]$GUI_CODE <- NA
```

Joined the mapping back to the NPNS data using:

7. FOOD CODE
8. COOKING METHOD

```
foodDataGUIMapped <-
  sqldf(
  "SELECT f.*, m.GUI_CODE  from foodDataForMapping f
  LEFT JOIN mappings m on m.FCODE = f.FCODE AND m.CMETH = f.CMETH"
  )
```

2. Non-covered analysis.Rmd.

Initially, the data was loaded after we mapped and the IUNA NPNS 77 Food Group was used to group the different foods.

The initial aggregation was done at `SUBJECT_ID` and `SURVDAY` survey day meaning that for each subject and each day of the survey we got an aggregated record.

We defined 4 aggregate metrics:

1. `non_gui_ct` number of entries in the diary which do not map to a GUI food code. Note that an entry in the diary is a *consumption*.
2. `non_gui_fwt` the total weight of the entries which do not map to a GUI food code.
3. `day_ct` total number of entries.
4. `day_fwt` total weight of the entries.

Note that all the 4 aggreates are agreggated at subject and survey day level, meaning that there is an entry for each subject and for each day of the survey.

```
foodDataGUIMapped <- read.csv("foodDataGUIMappedV2.csv")
foodDataSPSS <- read.spss(file="npns-food-file-4R.sav")

DOW <- c('SUN', 'MON', 'TUE', 'WED', 'THU', 'FRI', 'SAT')
foodDataGUIMapped$DOW <- factor(sapply(foodDataGUIMapped$DOW, functi
on(x){

    DOW[x]
}), levels = DOW)
# Loaded initially to map against the 77 food categories
iunaFoodCodes<- cbind.data.frame(code=seq(1,77), description=levels(fo
odDataSPSS$IUNA_NPNS_77FG))


nonGUIConsumptions <- sqldf("SELECT SUBJECID, DOW,
                            SUM(CASE WHEN GUI_CODE IS NULL THEN 1 ELSE 0 E
ND) non_gui_ct,
                            SUM(CASE WHEN GUI_CODE IS NULL THEN FWT ELSE 0
END) non_gui_fwt,
                            SUM(CASE WHEN GUI_CODE IS NULL THEN sugars ELS
E 0 END) uncovered_total_sugar,
                            SUM(CASE WHEN GUI_CODE IS NULL THEN 0 ELSE sug
ars END) covered_total_sugars,
                            SUM(sugars) total_sugars,
                            COUNT(*) day_ct,
                            SUM(FWT) day_fwt
                            FROM foodDataGUIMapped GROUP BY SUBJECID, DOW"
)
```

Ratio of non-GUI and total consumptions

Checked if the ratio changes over the day of the survey. Assumed independance across the 4 days.

Overall view

```
library(ggplot2)
nonGUIConsumptions$ratio_ct <- nonGUIConsumptions$non_gui_ct/nonGUIC
onsumptions$day_ct
nonGUIConsumptions$ratio_fwt <- nonGUIConsumptions$non_gui_fwt/nonGU
IConsumptions$day_fwt


par(mfrow=c(1,1))
xval <- nonGUIConsumptions$ratio_ct*100
h<-hist(xval, xlab='% of total count of consumptions per day',
     main="Unmapped consumptions ratio", col='orange')
xfit <- seq(min(xval), max(xval), length.out = 40)
yfit <- dnorm(xfit, mean = mean(xval), sd= sd(xval))
yfit <- yfit * diff(h$mids[1:2]) * length(xval)
lines(xfit, yfit, col='blue', lwd=2)
box()
```

```
xval <- nonGUIConsumptions$ratio_fwt*100
h<-hist(xval, xlab='% of total food weight per day',
     main="Unmapped food weight", col='orange')
xfit <- seq(min(xval), max(xval), length.out = 40)
yfit <- dnorm(xfit, mean = mean(xval), sd= sd(xval))
yfit <- yfit * diff(h$mids[1:2]) * length(xval)
lines(xfit, yfit, col='blue', lwd=2)
box()
```

```
## [1] "=====Uncovered Food Items Weight ====="

pastecs::stat.desc(nonGUIConsumptions[,c("non_gui_ct", "day_ct", "non_
gui_fwt", "day_fwt")])

##                 non_gui_ct       day_ct  non_gui_fwt      day_fwt
## nbr.val        504.0000000  504.0000000 5.040000e+02 5.040000e+02
## nbr.null         0.0000000    0.0000000 0.000000e+00 0.000000e+00
## nbr.na           0.0000000    0.0000000 0.000000e+00 0.000000e+00
## min              1.0000000    2.0000000 7.100000e+01 7.600000e+01
## max             21.0000000   35.0000000 1.307000e+03 2.466000e+03
## range           20.0000000   33.0000000 1.236000e+03 2.390000e+03
## sum           4057.0000000 9211.0000000 2.088480e+05 6.225530e+05
## median           8.0000000   18.0000000 3.900000e+02 1.214000e+03
## mean             8.0496032   18.2757937 4.143810e+02 1.235224e+03
## SE.mean          0.1349343    0.2223957 9.007997e+00 1.641964e+01
## CI.mean.0.95     0.2651043    0.4369389 1.769793e+01 3.225953e+01
## var              9.1764611   24.9277628 4.089658e+04 1.358807e+05
```

```
## std.dev        3.0292674    4.9927711 2.022290e+02 3.686201e+02
## coef.var       0.3763251    0.2731904 4.880268e-01 2.984236e-01
```

```
pastecs::stat.desc(nonGUIConsumptions[, c("ratio_ct", "ratio_fwt")])
```

```
##                  ratio_ct    ratio_fwt
## nbr.val      5.040000e+02 5.040000e+02
## nbr.null     0.000000e+00 0.000000e+00
## nbr.na       0.000000e+00 0.000000e+00
## min          1.250000e-01 4.899931e-02
## max          1.000000e+00 1.000000e+00
## range        8.750000e-01 9.510007e-01
## sum          2.238548e+02 1.738821e+02
## median       4.444444e-01 3.323618e-01
## mean         4.441563e-01 3.450041e-01
## SE.mean      5.476348e-03 6.785794e-03
## CI.mean.0.95 1.075933e-02 1.333199e-02
## var          1.511516e-02 2.320769e-02
## std.dev      1.229437e-01 1.523407e-01
## coef.var     2.768028e-01 4.415620e-01
```

Analysed how the ratio of the covered food varies over each day of the week.

First looked at the distribution of the total number of consumptions and the total food over the day of the week.1 = Sunday; 2 = Monday; 3 = Tuesday; 4 = Wednesday; 5 = Thursday; 6 = Friday; 7 = Saturday

```
library(sm)

library(boot)
par(mfrow=c(1,1))

nonGUIConsumptions$dowFactored <- factor(nonGUIConsumptions$DOW)

with(nonGUIConsumptions,
     list(
     ct=boxplot(day_ct~DOW, xlab="Day of the Week", ylab="Number of coms
umptions",
               main="Number of consumptions by day of the week - Count"),
     weight=boxplot(day_fwt~DOW, xlab="Day of the Week", ylab="Weight of
consuptions",
               main="Weight of consuptions by day of the week - Weight")
```

Then explored the actual ratio.

```r
with(nonGUIConsumptions,
     list(
     ct=boxplot(ratio_ct*100~DOW, xlab="Day of the Week", ylab="% of non-GUI Consumption (count)",
               main="% Uncovered foods by day of the week - Count"),
     weight=boxplot(ratio_fwt*100~DOW, xlab="Day of the Week", ylab="% of non-GUI Consumption (weight)",
               main="% Uncovered foods by day of the week - Weight")
     )
)
```

```r
reportedStats <- as.data.frame(aggregate(cbind(non_gui_ct, day_ct, non_gui_fwt, day_fwt, ratio_ct, ratio_fwt)~DOW, data=nonGUIConsumptions, function(x){
  rst <- pastecs::stat.desc(x)
  c(rst['nbr.val'], rst['min'], rst['max'], rst['range'], rst['median'], rst['mean'], rst['std.dev'])
} ))

kable(t(reportedStats[,-1 ]), col.names = reportedStats[,1 ], digits = 1, description="descriptive for key variable")
```

	1	2	3	4	5	6	7
non_gui_ct.nbr.val	88.0	63.0	58.0	64.0	63.0	68.0	100.0
non_gui_ct.min	2.0	2.0	2.0	3.0	1.0	2.0	3.0
non_gui_ct.max	18.0	19.0	16.0	16.0	19.0	18.0	21.0
non_gui_ct.range	16.0	17.0	14.0	13.0	18.0	16.0	18.0
non_gui_ct.median	8.0	8.0	8.0	8.0	9.0	8.0	7.0
non_gui_ct.mean	7.7	8.0	7.8	8.3	8.8	8.0	7.9
non_gui_ct.std.dev	2.8	3.2	2.8	3.0	3.6	2.8	2.9
day_ct.nbr.val	88.0	63.0	58.0	64.0	63.0	68.0	100.0
day_ct.min	2.0	3.0	7.0	10.0	7.0	7.0	8.0
day_ct.max	31.0	28.0	29.0	35.0	35.0	32.0	35.0
day_ct.range	29.0	25.0	22.0	25.0	28.0	25.0	27.0
day_ct.median	18.0	18.0	18.0	19.0	19.0	17.0	18.0
day_ct.mean	17.3	18.0	18.5	19.4	19.6	17.9	17.9
day_ct.std.dev	4.8	4.7	4.3	5.4	6.2	4.7	4.7
non_gui_fwt.nbr.val	88.0	63.0	58.0	64.0	63.0	68.0	100.0
non_gui_fwt.min	76.0	78.0	71.0	138.0	100.0	96.0	83.0
non_gui_fwt.max	1144.0	1307.0	1016.0	1154.0	980.0	1240.0	1291.0
non_gui_fwt.range	1068.0	1229.0	945.0	1016.0	880.0	1144.0	1208.0
non_gui_fwt.median	351.0	445.0	393.5	411.5	406.0	393.0	360.0

non_gui_fwt.mean	383.9	436.9	422.4	453.8	419.7	414.4	393.9
non_gui_fwt.std.dev	203.0	224.7	199.2	208.6	167.9	206.9	200.4
day_fwt.nbr.val	88.0	63.0	58.0	64.0	63.0	68.0	100.0
day_fwt.min	76.0	338.0	310.0	583.0	593.0	443.0	575.0
day_fwt.max	2452.0	2238.0	2260.0	2466.0	2329.0	1993.0	2224.0
day_fwt.range	2376.0	1900.0	1950.0	1883.0	1736.0	1550.0	1649.0
day_fwt.median	1127.0	1258.0	1259.5	1339.5	1222.0	1142.0	1182.0
day_fwt.mean	1147.9	1263.6	1331.8	1351.3	1249.1	1178.8	1193.6
day_fwt.std.dev	372.0	345.3	380.0	389.5	372.1	330.8	358.0
ratio_ct.nbr.val	88.0	63.0	58.0	64.0	63.0	68.0	100.0
ratio_ct.min	0.2	0.2	0.1	0.2	0.1	0.1	0.2
ratio_ct.max	1.0	0.7	0.8	0.8	0.7	0.8	0.8
ratio_ct.range	0.8	0.5	0.6	0.6	0.6	0.6	0.6
ratio_ct.median	0.4	0.4	0.4	0.4	0.5	0.5	0.4
ratio_ct.mean	0.5	0.4	0.4	0.4	0.5	0.4	0.4
ratio_ct.std.dev	0.1	0.1	0.1	0.1	0.1	0.1	0.1
ratio_fwt.nbr.val	88.0	63.0	58.0	64.0	63.0	68.0	100.0
ratio_fwt.min	0.1	0.1	0.0	0.1	0.1	0.1	0.1
ratio_fwt.max	1.0	0.8	0.7	0.7	0.8	0.7	1.0
ratio_fwt.range	0.9	0.7	0.7	0.6	0.7	0.6	0.9
ratio_fwt.median	0.3	0.3	0.3	0.3	0.3	0.4	0.3
ratio_fwt.mean	0.3	0.3	0.3	0.3	0.4	0.4	0.3
ratio_fwt.std.dev	0.2	0.2	0.1	0.1	0.1	0.1	0.2

A nonparametric density estimation was used and the distribution of the 4 variables investigated: 1. `day_ct` total count of consumptions 2. `day_fwt` total weight of food in a day 3. `ratio_ct` the ratio of the number of consumptions that are covered by GUI 4. `ratio_fwt` the ratio of the food weight of the consumptions that are covered by GUI

```
colfill <- c(2:(1+length(nonGUIConsumptions$dowFactored)))

sm.density.compare(nonGUIConsumptions$day_ct, nonGUIConsumptions$dow
Factored, xlab="food count" )
title("Distribution of food count by Day of the Week")
legend("topright", levels(nonGUIConsumptions$dowFactored) , fill=colf
ill)
```

```
sm.density.compare(nonGUIConsumptions$day_fwt, nonGUIConsumptions$do
wFactored, xlab="food weight" )
title("Distribution of food weight by Day of the Week")
legend("topright", levels(nonGUIConsumptions$dowFactored) , fill=colf
ill)
```

```r
sm.density.compare(nonGUIConsumptions$ratio_ct, nonGUIConsumptions$d
owFactored, xlab="Ratio of non GUI food count" )
title("Distribution of ratio of non GUI food count by Day of the Week")
legend("topright", levels(nonGUIConsumptions$dowFactored) , fill=colf
ill)
```

```r
sm.density.compare(nonGUIConsumptions$ratio_fwt, nonGUIConsumptions$
dowFactored, xlab="Ratio of non GUI food weight")
title("Distribution of ratio of non GUI food weight by Day of the Week")
legend("topright", levels(nonGUIConsumptions$dowFactored) , fill=colf
ill)
```

- **Test for ratio of count**

```r
plot(nonGUIConsumptions$DOW, nonGUIConsumptions$ratio_ct)
```

```r
print("==== RATIO CT === ")

## [1] "==== RATIO CT === "

ratioCtCoeff <- sapply(DOW, function(x, nonGUIConsumptions){
  xInclude <- nonGUIConsumptions[nonGUIConsumptions$DOW == x, 'ratio_
ct']
  xExclude <- nonGUIConsumptions[nonGUIConsumptions$DOW != x, 'ratio_
ct' ]

  xInclude<- sample(xInclude, 5000, replace = TRUE)
  xExclude<- sample(xExclude, 5000, replace = TRUE)
  print(sprintf("Permutation test = %s", x))
  print(wilcox.test(xInclude, xExclude))
}, nonGUIConsumptions)

## [1] "Permutation test = SUN"
##
##  Wilcoxon rank sum test with continuity correction
##
## data:  xInclude and xExclude
## W = 12866000, p-value = 0.01113
## alternative hypothesis: true location shift is not equal to 0
##
## [1] "Permutation test = MON"
##
##  Wilcoxon rank sum test with continuity correction
##
## data:  xInclude and xExclude
```

```
## W = 12470000, p-value = 0.8348
## alternative hypothesis: true location shift is not equal to 0
##
## [1] "Permutation test = TUE"
##
##  Wilcoxon rank sum test with continuity correction
##
## data:  xInclude and xExclude
## W = 10924000, p-value < 2.2e-16
## alternative hypothesis: true location shift is not equal to 0
##
## [1] "Permutation test = WED"
##
##  Wilcoxon rank sum test with continuity correction
##
## data:  xInclude and xExclude
## W = 11747000, p-value = 1.773e-07
## alternative hypothesis: true location shift is not equal to 0
##
## [1] "Permutation test = THU"
##
##  Wilcoxon rank sum test with continuity correction
##
## data:  xInclude and xExclude
## W = 13373000, p-value = 1.407e-09
## alternative hypothesis: true location shift is not equal to 0
##
## [1] "Permutation test = FRI"
##
##  Wilcoxon rank sum test with continuity correction
##
## data:  xInclude and xExclude
## W = 12913000, p-value = 0.00421
## alternative hypothesis: true location shift is not equal to 0
##
## [1] "Permutation test = SAT"
##
##  Wilcoxon rank sum test with continuity correction
##
## data:  xInclude and xExclude
## W = 12154000, p-value = 0.01647
## alternative hypothesis: true location shift is not equal to 0
```

```
print("=== DAY CT ==== ")
```

```
## [1] "=== DAY CT ==== "
```

```
ratioCtCoeff <- sapply(DOW, function(x, nonGUIConsumptions){
  xInclude <- nonGUIConsumptions[nonGUIConsumptions$DOW == x, 'day_ct
']
```

```
  xExclude <- nonGUIConsumptions[nonGUIConsumptions$DOW != x, 'day_ct
' ]

  xInclude<- sample(xInclude, 5000, replace = TRUE)
  xExclude<- sample(xExclude, 5000, replace = TRUE)
  print(sprintf("Permutation test = %s", x))
  print(wilcox.test(xInclude, xExclude))
}, nonGUIConsumptions)
```

```
## [1] "Permutation test = SUN"
##
##  Wilcoxon rank sum test with continuity correction
##
## data:  xInclude and xExclude
## W = 11038000, p-value < 2.2e-16
## alternative hypothesis: true location shift is not equal to 0
##
## [1] "Permutation test = MON"
##
##  Wilcoxon rank sum test with continuity correction
##
## data:  xInclude and xExclude
## W = 12590000, p-value = 0.5321
## alternative hypothesis: true location shift is not equal to 0
##
## [1] "Permutation test = TUE"
##
##  Wilcoxon rank sum test with continuity correction
##
## data:  xInclude and xExclude
## W = 13272000, p-value = 7.967e-08
## alternative hypothesis: true location shift is not equal to 0
##
## [1] "Permutation test = WED"
##
##  Wilcoxon rank sum test with continuity correction
##
## data:  xInclude and xExclude
## W = 14072000, p-value < 2.2e-16
## alternative hypothesis: true location shift is not equal to 0
##
## [1] "Permutation test = THU"
##
##  Wilcoxon rank sum test with continuity correction
##
## data:  xInclude and xExclude
## W = 14092000, p-value < 2.2e-16
## alternative hypothesis: true location shift is not equal to 0
##
```

```
## [1] "Permutation test = FRI"
##
##  Wilcoxon rank sum test with continuity correction
##
## data:  xInclude and xExclude
## W = 11774000, p-value = 4.618e-07
## alternative hypothesis: true location shift is not equal to 0
##
## [1] "Permutation test = SAT"
##
##  Wilcoxon rank sum test with continuity correction
##
## data:  xInclude and xExclude
## W = 11614000, p-value = 7.766e-10
## alternative hypothesis: true location shift is not equal to 0
```

Test for ratio of food weight

```
print("==== RATIO FWT=====")

## [1] "==== RATIO FWT====="

ratioCtCoeff <- sapply(DOW, function(x, nonGUIConsumptions){
  xInclude <- nonGUIConsumptions[nonGUIConsumptions$DOW == x, 'ratio_
fwt']
  xExclude <- nonGUIConsumptions[nonGUIConsumptions$DOW != x, 'ratio_
fwt' ]

  xInclude<- sample(xInclude, 5000, replace = TRUE)
  xExclude<- sample(xExclude, 5000, replace = TRUE)

  print(sprintf("Permutation test = %s", x))
  print(wilcox.test(xInclude, xExclude))
}, nonGUIConsumptions)

## [1] "Permutation test = SUN"
##
##  Wilcoxon rank sum test with continuity correction
##
## data:  xInclude and xExclude
## W = 12506000, p-value = 0.9688
## alternative hypothesis: true location shift is not equal to 0
##
## [1] "Permutation test = MON"
##
##  Wilcoxon rank sum test with continuity correction
##
## data:  xInclude and xExclude
## W = 12335000, p-value = 0.2539
```

```
## alternative hypothesis: true location shift is not equal to 0
##
## [1] "Permutation test = TUE"
##
##  Wilcoxon rank sum test with continuity correction
##
## data:  xInclude and xExclude
## W = 11531000, p-value = 1.93e-11
## alternative hypothesis: true location shift is not equal to 0
##
## [1] "Permutation test = WED"
##
##  Wilcoxon rank sum test with continuity correction
##
## data:  xInclude and xExclude
## W = 12841000, p-value = 0.01831
## alternative hypothesis: true location shift is not equal to 0
##
## [1] "Permutation test = THU"
##
##  Wilcoxon rank sum test with continuity correction
##
## data:  xInclude and xExclude
## W = 13089000, p-value = 4.479e-05
## alternative hypothesis: true location shift is not equal to 0
##
## [1] "Permutation test = FRI"
##
##  Wilcoxon rank sum test with continuity correction
##
## data:  xInclude and xExclude
## W = 14019000, p-value < 2.2e-16
## alternative hypothesis: true location shift is not equal to 0
##
## [1] "Permutation test = SAT"
##
##  Wilcoxon rank sum test with continuity correction
##
## data:  xInclude and xExclude
## W = 12023000, p-value = 0.0009522
## alternative hypothesis: true location shift is not equal to 0
```

```
print("=== DAY FWT ==== ")
```

```
## [1] "=== DAY FWT ==== "
```

```
ratioCtCoeff <- sapply(DOW, function(x, nonGUIConsumptions){
  xInclude <- nonGUIConsumptions[nonGUIConsumptions$DOW == x, 'day_fw
t']
  xExclude <- nonGUIConsumptions[nonGUIConsumptions$DOW != x, 'day_fw
```

```
t' ]

  xInclude<- sample(xInclude, 5000, replace = TRUE)
  xExclude<- sample(xExclude, 5000, replace = TRUE)

  print(sprintf("Permutation test = %s", x))
  print(wilcox.test(xInclude, xExclude))
}, nonGUIConsumptions)
```

```
## [1] "Permutation test = SUN"
##
##  Wilcoxon rank sum test with continuity correction
##
## data:  xInclude and xExclude
## W = 10519000, p-value < 2.2e-16
## alternative hypothesis: true location shift is not equal to 0
##
## [1] "Permutation test = MON"
##
##  Wilcoxon rank sum test with continuity correction
##
## data:  xInclude and xExclude
## W = 13713000, p-value < 2.2e-16
## alternative hypothesis: true location shift is not equal to 0
##
## [1] "Permutation test = TUE"
##
##  Wilcoxon rank sum test with continuity correction
##
## data:  xInclude and xExclude
## W = 15005000, p-value < 2.2e-16
## alternative hypothesis: true location shift is not equal to 0
##
## [1] "Permutation test = WED"
##
##  Wilcoxon rank sum test with continuity correction
##
## data:  xInclude and xExclude
## W = 14859000, p-value < 2.2e-16
## alternative hypothesis: true location shift is not equal to 0
##
## [1] "Permutation test = THU"
##
##  Wilcoxon rank sum test with continuity correction
##
## data:  xInclude and xExclude
## W = 12737000, p-value = 0.1005
## alternative hypothesis: true location shift is not equal to 0
##
```

```
## [1] "Permutation test = FRI"
##
##  Wilcoxon rank sum test with continuity correction
##
## data:  xInclude and xExclude
## W = 11359000, p-value = 2.707e-15
## alternative hypothesis: true location shift is not equal to 0
##
## [1] "Permutation test = SAT"
##
##  Wilcoxon rank sum test with continuity correction
##
## data:  xInclude and xExclude
## W = 11247000, p-value < 2.2e-16
## alternative hypothesis: true location shift is not equal to 0
```

- **Daily intake and Snacks**

```
missedFoodAvgDailyIntakeBySubjects <- sqldf("SELECT SUBJECID, IUNA_NPN
S_77FG,
                              CASE WHEN MTYPE IN (6,7,8,11) THEN 'Snack' E
LSE 'Main meal' END meal_type,
                              SUM(CASE WHEN GUI_CODE IS NULL THEN 1 ELSE 0
END)/4.0 non_gui_fq_daily_avg,
                              SUM(CASE WHEN GUI_CODE IS NULL THEN FWT ELSE
0 END)/4.0 non_gui_fwt_daily_avg
                              FROM foodDataGUIMapped
                              GROUP BY SUBJECID, IUNA_NPNS_77FG,
                              CASE WHEN MTYPE IN (6,7,8,11) THEN 'Snack' E
LSE 'Main meal' END
              ", verbose = TRUE)

missedFoodDailySummary <- as.data.frame( as.matrix(
  aggregate(non_gui_fwt_daily_avg~IUNA_NPNS_77FG+meal_type, data=mis
sedFoodAvgDailyIntakeBySubjects, function(x){c(mean(x), sd(x), quant
ile(x,0.50), IQR(x))})
  ))
colnames(missedFoodDailySummary) <- c('IUNA_NPNS_77FG', 'meal_type', '
avg_di', 'sd_di', 'p50_di', 'iqr_di')


exportSummary <-
merge(
missedFoodDailySummary[missedFoodDailySummary$meal_type == 'Snack', c
(1,3,4,5,6)],
missedFoodDailySummary[missedFoodDailySummary$meal_type != 'Snack', c
(1,3,4,5,6)],
by= "IUNA_NPNS_77FG",
```

```
suffixes = c("_snack", "_main_meal"),
all=TRUE
)

kable(exportSummary, caption = "Summary stats using daily intake")
```

stats using daily intake

- **Consumption averages**

```
missedFoodMeanIntakeBySubjects <- sqldf("SELECT SUBJECID, SURVDAY, IUNA
_NPNS_77FG,
                              CASE WHEN MTYPE IN (6,7,8,11) THEN 'Snack' E
LSE 'Main meal' END meal_type,
                              SUM(CASE WHEN GUI_CODE IS NULL THEN 1 ELSE 0
END) non_gui_fq_daily_avg,
                              AVG(CASE WHEN GUI_CODE IS NULL THEN FWT ELSE
0 END)  non_gui_fwt_daily_avg
                              FROM foodDataGUIMapped
                              GROUP BY SUBJECID, IUNA_NPNS_77FG, SURVDAY,
                              CASE WHEN MTYPE IN (6,7,8,11) THEN 'Snack' E
LSE 'Main meal' END
              ")


missedFoodMeanSummary <- as.data.frame( as.matrix(
  aggregate(non_gui_fwt_daily_avg~IUNA_NPNS_77FG+meal_type, data=mis
sedFoodMeanIntakeBySubjects, function(x){c(mean(x), sd(x), quantile(
x,0.50), IQR(x))})
))
colnames(missedFoodDailySummary) <- c('IUNA_NPNS_77FG', 'meal_type', '
avg_di', 'sd_di', 'p50_di', 'iqr_di')


exportMeanSummary <-
  merge(
    missedFoodMeanSummary[missedFoodMeanSummary$meal_type == 'Snack',
c(1,3,4,5,6)],
    missedFoodMeanSummary[missedFoodMeanSummary$meal_type != 'Snack',
c(1,3,4,5,6)],
    by= "IUNA_NPNS_77FG",
    suffixes = c("_snack", "_main_meal"),
    all=TRUE
  )

kable(exportMeanSummary, caption = "Summary stats using consumption aver
ages")
```
